# Higher education and the COVID-19 pandemic: navigating disruption using the sustainable development goals

**DOI:** 10.1007/s43621-021-00013-2

**Published:** 2021-02-16

**Authors:** Wendy M. Purcell, Julio Lumbreras

**Affiliations:** 1 Harvard T.H. Chan School of Public Health, Harvard University, 401 Park Drive, 4th Floor West Suite 406, PO Box 15677, Boston, MA 02215 USA; 2 Innovation and Technology for Development Centre, Universidad Politécnica de Madrid (itdUPM), Department of Chemical Engineering and Environment, Industrial Engineering School, Universidad Politécnica de Madrid, Madrid, Spain

## Abstract

The COVID-19 health crisis has caused profound social and economic disruptions. Affecting everyone, its impact is not equal. Exacerbating deep social fissures and long-standing systemic inequalities, the pandemic reveals the fragilities and inequities in global higher education. Accelerating underlying trends and triggering seismic shifts in the sector, collective efforts over a period of weeks delivered massive change in a sector not known for being fleet-of-foot. As we envisage the ‘next normal’ for higher education, problems with the ‘old normal’ may be fixed and some recent innovations carried forward. Representing a period of punctuated equilibrium, COVID-19 could lead to transformation of the sector towards greater equity and impact across teaching/learning, research/innovation, community service/engagement and the staff/students’ experience. As we ask, “When will HE open again?”, and “When we do, what will it be like?” we may also find ourselves considering “Will some institutions open at all?” Seeking to navigate these volatile, uncertain, complex and ambiguous times—so-called VUCA conditions—we propose that the SDGs are positioned as a lens to re-imagine higher education in mounting an antifragility response. We examine whether a new paradigm is forming that could be more sustainable as budgets, priorities and institutional archetypes are challenged fundamentally in line with delivery against the SDGs.

## Introduction

The health, social and economic crises of the COVID-19 pandemic have thrown into sharp relief the disruptive forces acting on the global higher education (HE) sector [1, 2]. For example, at the beginning of March 2020 in the United States of America (US), no one imagined HE would change so drastically. By mid-March lockdowns and physical distancing strategies effectively closed campuses. Most institutions in the US pivoted quickly to online delivery as a temporary measure, a move echoed around the world. The Class of 2020 went on to complete online, graduate virtually and enter a challenging job market. With refunds on tuition, room and board and loss of auxiliary income from endowments, philanthropy, conferences, athletics and research, the US HE business model is under threat [3, 4]. For example, the University of Wisconsin estimated a US $170M loss in spring 2020 from refunding room, dining and parking alone, while Bucknell University’s endowment of US $867M lost US $150M and Harvard University’s distribution from endowment income comprising 35% of its operating budget lost some 30% [5]. Some one in five US colleges are deemed to be at risk [6] given severe budget pressures. Those in Europe and elsewhere are somewhat shielded by government-backed tuition loans and stimulus packages [7, 8]. During the pandemic, HE institutions worldwide responded at speed and delivered massive change in a manner of weeks as they reacted to the COVID-19 challenge. The ‘patient’ survived, but will it now thrive? And, can the Sustainable Development Goals (SDGs) help accelerate transformation of the sector to the ‘next normal’? Here we examine the impact of the pandemic across a number of different activities in HE and highlight how the SDGs can be used to help the sector navigate the disruption.

Higher education serves a wide variety of stakeholders through its functions of education, research and service; most are important local employers and drivers of local and regional economies, many are civic institutions—operating as small cities with hospitals, science parks and innovation centers part of the campus. Through these functions, HE contributes directly to delivery against the targets in SDG1 (poverty), SDG3 (health and well-being), SDG4 (education), SDG5 (gender equality), SDG8 (decent work and economic growth), SDG12 (responsible consumption and production), SDG13 (climate change), SDG16 (peace, justice and strong institutions) and features in many ‘partnerships for the goals’ (SDG 17) [9]. Given the complexity of the US revenue model (tuition fees, government/state grants, philanthropic donations, and income from endowments, sales and services) and changes to applicant behavior (reduced enrollments, higher non-continuance etc.), most US HE institutions are experiencing enormous financial unpredictability [4]. The wider forces of disruption due to COVID-19 and other global megatrends will affect the business model for HE, institutional priorities and overall mission. How might this affect HE’s important contribution towards achievement of the SDGs? As we ask “When will universities and colleges open again?” and “What will it be like when we do?” some institutions may be asking, “Will we open at all?”

The pandemic therefore represents an inflection point for HE—accelerating underlying trends and exposing fissures and inequities. It is drawing attention to the interconnected and hyper-dependent nature of universities and colleges with the public, private and plural sectors. Looking ahead, the financial challenges and behavioral changes among stakeholders could ultimately decimate the sector. More likely, HE will be reshaped fundamentally [10], with COVID-19 representing a ‘teachable moment’ for the sector—a period of punctuated equilibrium rather than the evolutionary gradualism more typically associated with change in HE. As a sector, HE is subject to the same responses to disruption as other sectors, for example, new entrants, the dominance of the incumbents for a period, new regulations and mergers and acquisition [11, 12]. This is the moment for the sector to highlight its central role in recovery and transformation of the economy and society ahead, taking up its position in helping create a more equitable and sustainable future through fulfilment of the SDGs [13]. How might we sustain the role universities and colleges play as anchor institutions in place making, contributing to economic development and community service? How might we continue to drive HE’s role in convening private, public and third sector actors to capture the enormous potential HE offers to deliver the SDGs [14]. With a focus on how we might ‘build back better’ and embrace antifragility [15], we explore here the impact of COVID-19. We look at how the sector *reacted* and then *responded*, moving forward to *reimagine* and *renew* itself to frame the ‘next normal’ around Agenda 2030 [16].

The future of HE will rely on bold persistent experimentation. Reacting to the current disruptive and challenging times creates a space for HE to respond more deliberately. For example, the pivot from high-touch to low- or no-touch pedagogy will demand we re-examine pedagogy and programs, while working-from-home may mean we will look again at planned multi-million dollar campus building projects and invest more in a digital strategy. With some 5000 HE institutions in the US alone, the different archetypes—from 4-year private to 4-year public universities, community colleges and mega-universities, will be affected differentially by the pandemic. This points to an increased differentiation in the US sector, as universities and colleges focus on income earned through value created. In some US states, we may witness the decline of the college town, ‘no gown, to ghost town’, with anchor institutions role in place-making challenged [1]).

COVID-19 effectively pathologized the closeness and collegiality of an academic community, something the ‘Zoomiverse’ cannot recreate. For some US institutions, empty dorms equate to an empty balance sheet [17], with refunds of tuition fees, room and board payments translated to a freeze on hiring, staff furloughs and layoffs, and a ‘gig academy’ [18, 19] with precariat faculty, a tenure clock on pause, more part-time and non-tenured positions and reductions in benefits. While the pandemic is an opportunity to take a critical look at HE, we are already seeing sharing, merging and take-overs proposed as a solution to tackling the issues in the sector [20]. The pandemic may accelerate efforts to consolidate the sector, with drivers and obstacles more starkly presented. Indeed, in a survey of US Chief Business Officers in HE, some 50% said they plan to use this period to make difficult but transformative changes to structure and operations [21]. How could the SDGs influence their thinking to make decisions for the longer-term? During the crisis, HE worked with other stakeholders in their cities and beyond, opening up their physical space and offering access to resources to help local governments and health systems tackle COVID-19. From early release of health and medicine graduates to 3-D printing of personal and protective equipment, the HE sector leaned into the crisis [22, 23]. Is this a one-time reaction to the pandemic or could some of these practices be incorporated into the daily functioning of HE institutions to deliver against the SDGs and the global challenges the humanity is facing?

Here we explore how the HE sector *reacted* to the COVID-19 situation as people triaged its unfolding impact. We go on to reflect on its more considered *response,* as the sector moved to pivot and begin its recovery. Finally, we share some reflections on the lasting transformational impact on institutional priorities for sustaining the sector as it *re*-*imagines* itself. We posit that the SDGs represent a shared purpose around which HE can gather to make sense of and help navigate the disruptions caused and/or revealed by the pandemic.

## Methodology

We reviewed the public strategies undertaken by US HE in particular, and undertook discussions with some Presidents and senior team members across the sector between April 2020 through September 2020, together with discussions at the United Nations High-level Political Forum [26, 27]. We also drew upon a closed conversation with a panel of seven university and college Presidents undertaken under the auspices of the Massachusetts Women’s Forum (MWF), with the event opened up to the global audience under the International Women’s Forum (see Box 1). The term ‘President’, as it relates to a US university/college is co-terminus with that of a Rector in a European university, a Vice-Chancellor in a UK university, and a Chief Executive Officer (CEO) in terms of their executive administrative responsibilities for strategy, character and mission of the higher education institution concerned. While they may perform ceremonial duties, such as presiding over graduation ceremonies or conferring honorary degrees, they are executive in terms of the functions they perform. Within the formal governance structure, the President would typically report to a formal Board comprising external (lay) people; the board that may also include faculty, staff and students.

The seven different US HE institutions were a convenience sample drawn from the membership of MWF in response to an open call by the Director. They represent a diversity of institutional types in the Commonwealth of Massachusetts. This state has 114 colleges and universities listed under the Carnegie Classification of Institutions of Higher Education, 85 of which are private with five for-profit, and include 14 research universities, 21 master’s universities and 34 special focus institutions. Given the source of the sample, all the Presidents were women. Simmons University is a private university with around 7000 students and offers a women’s only undergraduate program and co-education graduate programs; it began in 1899 and secured university title in 2018. Wellesley College is an elite high-rank private women’s liberal arts college established in 1870 and has under 3000 students. Regis College is a private Roman Catholic university founded in 1927 for women, but has admitted men since 2007; it has around 3000 students following programs across arts, sciences and business. Cambridge College is a private non-profit institution offering adult learning programs; it has 3000 students and was opened around 45-years ago to offer a range of certificate, degree and masters programs. Greenfield and Roxbury are two of Massachusetts 15 public not-for-profit two-year community colleges; Greenfield was set up in 1962 to serve a largely rural population of adult learners, while Roxbury opened in 1973 to support an inner-city population of adult learners with a majority minority population. Bentley University, founded in 1917, is a private for-profit institution focused on business programs with around 6000 students. Given the different institutional archetypes, the methodology as it relates to the discussions with the seven US presidents under the auspices of the MWF may be considered a case study approach given the usefulness of this method for obtaining an in-depth appreciation of an issues or area of interest in its natural real-life context. While comparison among universities/colleges is difficult, the aim was to understand the influence of COVID-19 on the institution and explore how the SDGs might help navigate the disruption caused by the pandemic.

Box 1: Online panel discussion with University/College Presidents in Massachusetts, US The Massachusetts Women’s Forum (https://masswomensforum.org/) hosted a virtual roundtable event on 12 May 2020 to explore the ‘Impact of COVID-19 on the Higher Education Sector’, with the session opened up to a global audience through the International Women’s Forum (https://www.iwforum.org/). Joined by distinguished university and college Presidents^1^ from seven very different institutions across the Commonwealth of Massachusetts, author Wendy Purcell designed and moderated the discussion and captured learning insights from the session [28]. COVID-19 is challenging everything we do in universities and colleges, from teaching and learning, research and innovation, the students’ experience, faculty and staffing levels, investments in technology and student support, infrastructure projects, fund-raising, internationalization efforts and so much more. With over 100 colleges and universities in the Commonwealth of Massachusetts listed under the Carnegie Classification of Institutions of HE, including 14 research universities, 21 master’s universities, and 34 special-focus institutions, the panel reflected the diversity of HE missions and contexts. We often refer to a global HE sector, but fail to acknowledge fully its distinctiveness and differentiation. From community colleges to liberal arts universities, those focused on adult learners, others offering an immersive on-campus experience, women’s colleges, those in urban or rural locations, public and private sector; each will react and respond to the COVID-19 challenge in line with their vision and strategic priorities, as well as financial health. As we explore the impact of the pandemic and the sector’s response to the disruption of lockdown and its downstream economic impacts, there is no one-size fits all approach to tackling the challenges now and ahead for HE.

The pandemic is affecting the sustainability of the HE sector, revealing its fragilities and highlighting issues of equity in our system. Helen Drinan noted, *We entered this threatening period on a lot of weak notes.* As these seismic shifts re-shape higher education, the panel shared their views on what it will take to re-imagine a ‘new normal’ for the sector. Commenting on the incredible agility shown in moving online in a matter of weeks, Helen Drinan said this tells us *If we want to change, we can change.* As the longer-term implications play out, it was clear these institutional leaders were thinking deeply about the profound disruption in, and to, the sector. As we move now into the recovery ahead, Wendy Purcell explored *When and how we will open?* and reflected on whether we should also ask *Will we open at all?*

Paula Johnson captured the mood of the discussion, saying, *This is a moment of crisis and also of tremendous opportunity*, highlighting how the COVID-19 crisis had laid bare tremendous inequities in society as well as the opportunity to build back better. Toni Hays developed the theme of equity and called on HE to be made *more affordable, more accessible, more efficient and more collaborative.* Deborah Jackson reminded us of the critical role of HE in opening doors to opportunity, reminding us that in sending students home some of them are *not a child going home to parents, rather they are the parents.* Going on describe the particular concerns of adult learners at Community Colleges in low-wage, low-benefit frontline and service jobs at risk from the pandemic lock-down, Deborah Jackson outlined the targeted support offered from Wi-Fi hotspots to providing access to computer labs. Highlighting the importance of campus as home and campus as community, Paula Johnson emphasized the importance of HE and diversity as essential to our democracy. Taking the discussion on, Yves Solomon-Fernandez underlined the special needs of those in rural communities where lack of access to transport and Wi-Fi were major issues. Yves Solomon-Fernandez forecast, *The world to which we will return will be dramatically different.* and called on us to *Chart a brighter and better path forward.*

Helen Drinan reminded us all that *Hope is not a strategy* and pointed to the work ahead, representing some of the most difficult leadership decisions any President in the sector will face in a lifetime. Drawing attention to the tensions between pricing and online/on-campus delivery of teaching, Helen Drinan was emphatic that *Tuition must fall.* Alison Davis-Blake demanded that we *Improve productivity in higher education,* noting the need to both invest and disinvest and learn from other sectors about process improvement. Alison Davis-Blake went on to describe *creative destruction*, reminding us to *find the intersection of mission and market*. Deborah Jackson agreed, stating it was *Hard to justify the cost of higher education today* calling on the sector to review the way we teach and learn—both the how and the what, noting that online learning is a strategic priority and is not the correspondence course of old. Valerie Roberson drew attention to the important role of HE in recovery of the economy, noting that when unemployment is high more people enter education and called on us to *support the new economy after the crisis.* While it has been said that ‘Forecasting is hard—especially the future!’, one thing is sure, the only certainty for HE is more uncertainty. Nevertheless, if the far-sightedness and determination of the assembled Presidents is any guide to the high caliber of leadership in the sector, we can feel confident we are safe; closing the session, Wendy Purcell offered a quote to guide the way:

*“Start by doing what’s necessary. Then do what’s possible. And, suddenly you’re doing the impossible.”*

Francis of Assisi

## Results

We explored the responses by the higher education sector to the disruption caused by the COVID-19 pandemic, directly and indirectly, with a focus on the US in particular. We identified eight domains of material interest, noting pre-COVID-19 conditions and contrasting these with innovations fueling the transformation to the ‘next normal’. Table 1 highlights key observations, with each domain discussed further in the following sections.

**Table 1:** Responses by the Higher Education sector to the disruption caused by the COVID-19 pandemic that might fuel transformation of the sector over the longer-term towards embracing the SDGs

Domain	Pre-COVID-19 conditions	Innovations fueling transformation
1. *Leadership* Dewar et al. [25] Marcero [12] Purcell [29]	Top-down, command-control Telling style of leadership communication Disrupted by global megatrends Inequities tolerated	Adaptive with social networks Invitational dialogue & reciprocity Focus on institutional purpose Pursuit of equity
2. *Financials* Huron [20] Marcero [12] Taparia [31]	Weakened financials & dependence on fees income Investment in physical assets Research supported by cross-subsidy from other income Administrative bureaucracy	Focus on income earned through value created & diversification of income Investment in digital & knowledge assets Research focused on scholarly inquiry & societal value Primacy of scholarly community
3. *Campus* Brown and Kafka [37] Lanich [36]	Campus as home Safety & security	Scholarly community campus & digital Health & well-being
4. *Internationalization* Dickler [49] Horne [48]	Focus on student mobility Dominance of host-country pedagogy	Focus on global citizenship Pedagogy of encounter & inter-cultural learning
5. *Teaching and Learning* Gates [42] McMurtrie [55] Smith 2016 [11] Taparia 2020 [31]	Scholarship for employability Opportunity gap persists & held as achievement gap Dominance of face–face pedagogy Synchronous learning	Scholarship for societal impact Focus on equity: diversity, inclusion & belonging Blended learning Synchronous & asynchronous delivery
6. *Employability* Strada Education Network [41] Willetts [59]	Privileged individualism & competition Career-focus Job skills & competencies	Focus on social justice & community Employability throughout life Life skills & values
7. *Sustainability* SDSN [65] SDSN [66] THE [62]	Environmental sustainability Operational focus Disconnected from formal curriculum	Sustainable development Strategic agenda Education for sustainable development within formal curriculum
8. *Multi-stakeholder Partnerships* Caplan et al. [69] Horan 2019 [71] Mataix et al. [68] Moreno-Serna et al. [70]	Ivory tower Knowledge transfer Operational/tactical partnerships Pursuit of mission	Connected university Knowledge exchange Strategic partnerships Multi-stakeholder assembly convened around shared societal purpose

### Leadership

Even before the pandemic, HE leaders in the US faced a difficult reality: financial problems, campus conflict, and intense disquiet over fees, debt and return on investment/qualifications at the level of the student and society [12]. They entered the crisis challenged by a range of megatrends from demography, opportunity gaps revealing inequities in student outcomes, lack of faculty diversity, and growing public concerns about rising tuition fees and unemployed and/or under-employed graduates. COVID-19 exacerbated many of these risks and added new threats to the business model, brand and civic responsibilities of universities and colleges. While most recognize any crisis presents both danger and opportunity, only the most cynical leaders seized the moment and reacted with early announcements of program closures and faculty lay-offs. Most however took a more measured approach, taking time to respond to the situation with people improvising in the moment of the crisis but with a view on shaping the new order. Concerned with reopening in the fall, their decisions had potentially life-or-death consequences [24]. This requires a combination of crisis (reactive) and strategic (responsive) decision-making, with an innovation team riding alongside the business recovery team to capture novel practices [25]. Navigating the crises hitting the sector, it is imperative that actions over the short-term pay all due cognizance to securing the longer-term and it is here that positioning the SDGs at a strategic level can influence decision-making.

The pandemic revealed that crisis planning and training was not a routine risk management strategy in HE, with most institutions assembling people into ad hoc groups to react to the immediate and largely operational demands of the pandemic. Seeking to manage using closed, centralized groups however fails to include the knowledge and insights available from wider networks and the lived experience of students, faculty and staff. However, if such networks are too loose they may not be able to act fast enough or may lack the authority to act on incomplete information [29]. Those that sought to lead their way through the uncertainty behaved in different ways, dealing with the immediate challenges with a view to the wider impact of their responses on student success, faculty engagement and staff well-being. Being invitational, leaders learned about the particular impact of the COVID-19 crisis on individuals and tailored their responses accordingly. Rather than one-directional communication, leaders engaged in dialogue within their institutions and included a wider circle of alumni, civic and business leaders and peers in other HE settings. Leveraging these networks enabled strategic investments alongside efficiency and effectiveness measures, driving budget adjustments, with leaders sensitive that reactive de-costing can cause longer-term de-valuing. Those who simply reacted to the crisis as a financial calamity acted accordingly over the short-term. While it is too early to discern response patterns among senior leaders, some trends relating to their behaviors are emerging (see Table 2).

**Table 2:** Reactive versus responsive leadership behaviors during COVID-19 in the Higher Education Sector

Illustrative senior leadership attributes	
Reactive	Responsive
Manage	Lead
Telling	Asking
Communication	Dialogue
Planning	Learning
Silos-Hierarchy	Networks
De-valuing	Investing
Strategy	Purpose

Recognizing that COVID-19 is a human crisis first, saw the majority of leaders lead from a human perspective, with empathy and compassion, developing a shared awareness of institutional fragilities and strengths. Prioritizing physical and psychological safety of students and staff, leaders responded to the crisis by paying attention to ensuring delivery aligned with institutional values—igniting hope that the future can be re-imagined for the university or college concerned and contribute to a more sustainable society. A focus on institutional purpose allowed leaders to flex and pivot their underlying strategies powerfully to the actual, perceived and expected risks presented by and/or revealed through the pandemic; improvisation was easier when there is a strong mission and vision in place with a clear sense of institutional identity. Bringing an equity lens to every decision, many of the actions HE leaders took recognized the differential vulnerability of people and they tailored their responses accordingly; other reacted with a one-size fits all approach that failed to fully recognize the impact of the situation on individuals.

### Financials

After the Great Recession of 2008, tuition fees in the US rose each year by an average of 4%, up some 40% over the period 2009–2019; this is not an option for most US HE institutions seeking to bridge the funding gap after COVID-19. Given the economic impact of the pandemic on family income and employment levels in the US and societal concerns about student loan debt—currently US $1.6TN in the US [30], there is low tolerance for fee rises and indeed huge pressures to make HE more affordable [31]. Declining revenue streams and escalating cost structures had ratings agencies, such as Moody’s, S&P and Fitch Sector Outlooks, rate the sector as ‘negative’. Student enrollment declines of 5–20% were expected for many US universities and colleges in fall 2020, with 5–13% declines in net tuition and auxiliary revenues per student in fiscal year 2021 [20]. With strategies for income-generation in most US HE institutions constrained by the macro-economic climate, many will therefore turn to de-costing. Given labor accounts for around 70% of institutional budgets in the US, we can expect material restructuring of faculty and staffing levels [19]. Most US HE universities and colleges entered the COVID-19 crisis in a weakened financial state, and had under-invested in online learning, their digital strategy and delivering structural change. Some 650-plus US HE institutions had experienced enrollment declines of 5% or more in the five years prior to the pandemic and 11 institutions announced plans to close or declared exigency between mid-March and mid-July 2020 [20]. With administrative spend outpacing faculty hiring, we can only hope that this is a moment for HE to reduce bureaucracy and focus on supporting value-creating faculty while increasing investment in their digital strategy. Joint ventures and shared service agreements may be relevant turnaround strategies [12].

The economic fallout from COVID-19 for universities and colleges, as for many sectors of the economy, will be unprecedented, its overall impact on HE finances being potentially deeper and longer than the 2008 economic crisis. In the US, some 800 HE institutions are predicted to lose 20% of their income with one in five US colleges at risk of closing [32, 33]. Revenue is predicted to be down significantly for most institutions in the coming year, a unique combination of fewer students entering and/or returning in fall 2021, including dramatic changes to international student mobility, with endowments and philanthropy challenged by the economic shocks due to the pandemic. Significant lost income has come from the waiving of accommodation fees for students, loss of catering and conferencing income with increased spending on technology and student support. Impacts in the current financial year differ across institutions, ranging from relatively minimal to tens of millions of US$ for some institutions with potential losses to income having multi-year impact. For the 2020/21 academic year, annualized international student fee income of around US $8BN is at risk in the UK alone [34], while in Australia, where the beginning of the virus coincided with the start of the university term, the International Education Association warned of a US $4-6BN hit if Chinese students could not attend the first term [35]. HE is looking to access one of the many schemes governments worldwide have introduced, using revolving credit facilities and/or securing longer-term funding through their banks, bonds or private placement markets. In the US, the Federal Government included higher education in the Coronavirus Aid, Relief, and Economic Security (CARES) Act [5]), offering some US $12.5BN to support the HE sector with half the funds going directly to students.

Research in HE is typically cross-subsidized by other income, recovering only 70% of indirect costs, so will demand careful stewardship in order to preserve the centrality of universities and colleges to our global knowledge economy, and to offer sustainable pathways for all the economic sectors. As the crisis continues, the research-focused models of many universities and colleges will come under strain given that tuition fees and/or endowment income is often used to subsidize research costs. A driver for more collaboration, costs-sharing and targeted philanthropy, we anticipate faculty will set their sights on making a bigger contribution to fulfilment of societal challenges represented by the SDGs. We are already seeing major foundations in the US re-orient their funding strategy toward equity and a world where no-one is left behind, e.g. the Robert Wood Johnson Foundation.

### Campus as home

In the US, sending students and staff home, while an appropriate reaction to the immediacy of the COVID-19 threat, served to reveal many of the inequities a ‘campus as home’ model seeks to attenuate, being key SDG targets such as equity. Even for non-residential colleges and universities, access to learning resources and social spaces create safe environments and a scholarly community. For some students and indeed staff, there was no home to return to, or rather no safe home—with some facing threatening environments and risk of abuse. Others struggled with accessing the internet, either lacking the technology required and/or connectivity, with no quiet place to study; most libraries, cafes and other public spaces in the US were closed during the lockdown, exacerbating the situation. Some colleges and universities responded by providing devices to students, for example, Ivy Technology Community College shipped mobile hotspots to people, issued 700 laptops and extended campus Wi-Fi to their car parks [36]. Insecurities around food, housing, jobs and health services all served to create high anxiety levels among current college and university students with mental health a growing crisis [37] as people struggle to deal with the shock and social isolation of the situation. A more conscious response demands that student needs beyond the classroom are accommodated, with wraparound services to support academic and personal success with efforts to re-create peer support and the vibrancy of campus life online. Moreover, ways to extend activities that add value to student life on campus similarly enrich the digital learning space. For example, inviting stakeholders to engage with curious minds in co-creation and collaborative work and adopting a problem-based methodology to support deep learning [38] can model the local and global partnerships required to deliver the SDGs.

As the pandemic subsides, most US HE institutions signaled their intention to return to campus in fall 2020—but many are already walking back their plans [33, 39, 40]. For example, the University of North Carolina at Chapel Hill told students to return home just one week after returning to campus, while Michigan State University advised students who had planned to live in residence halls to stay home. The first priority being the health and safety of students and staff, the dominant model for fall is online, with some offering an on-campus experience for some classes and/or programs, such as laboratory sessions [6]. However, tuition levels for online education have typically remained at on-campus prices—which many view as untenable, with calls for unbundling of instruction from other activities. Earlier in the year, one in six college bound high school students were rethinking whether to pursue HE in fall 2020 [41], with high unemployment and financial uncertainty meaning that many families may not able to support students and self-funded students will be similarly challenged, so enrollment is predicted to decrease. With reduced state support for US HE, some institutions may not be able to weather this storm and will be ‘at risk’. Typically, in recessionary times, more people enter US HE—with HE enrollment countercyclical with jobs, with increased admissions within 12–18 months of an economic downturn [42]. Given the economy was repressed deliberately to deal with the health crisis of the pandemic, it is unclear whether this will be the response this time around. A survey in August 2020 revealed some 40% of prospective students in the US planning to enroll at a four-year residential college were likely or highly likely not to do so, with almost one in three returning students planning not to go back or yet to decide [21]. This was the case even for the more prestigious and/or competitive institutions, for example Harvard University commented that over 20% of first year students deferred enrollment and between 80 and 110 students took a gap year [43, 44].

### Reimagining Internationalization

Universities and colleges reacted promptly to support the welfare of international students during the pandemic, which presented some specific challenges; some international students were stranded, unable to return home as countries closed to incoming flights—changes to visas regulations, work-authorization rules and border controls exacerbated the situation in the US. There is still so much ambiguity to navigate, with rising concerns over racist behaviors and nationalist commentary in some destinations [45, 46], as well as ongoing visa challenges to access in-country what are largely online programs [47]. This is challenging the academic and financial contributions international students make to the HE sector, with some institutions and countries overly reliant on international revenue [48]. In the US, new rules for international students could cost US colleges US $41 billion [49], while a 10% fall in enrollments from eastern Asia this fall could cost U.K. universities US $280 M in lost tuition fees alone [34]. In Australia, universities are estimated to lose around US $2.2–3.4 billion in revenue from international student fees in 2020, and more in 2021 [48].

The pandemic may represent a watershed for internationalization with global HE changed forever. How might we re-think, re-design, and overall re-imagine internationalization in a non-mobile world—or at least one where international travel is severely restricted? Internationalization has been taken to be coterminous with global mobility and the presence of international students, and indeed faculty, on campus. However, this is a narrow view and fails to appreciate the importance of global connections facilitated through teaching, research and service. Global learning—how we engage students with the complex inter-dependent and hyper-connected world we inhabit—and enable them to become global citizens and “SDG implementers” [50] demands we re-consider a pedagogy of encounter and design curriculum and learning journeys to encourage inter-cultural competencies. We need to re-create global communities of learning and connect our universities and colleges to the people HE serves. Creating a truly internationalized learning experience demands curricula reflect international examples and circumstances, rather than being dominated by domestic considerations, supporting the development of a global mindset and worldviews. For instance, EELISA is a European HE Consortium that is defining and implementing a common model of a European engineering curriculum rooted in society to implement the SDGs [51]. International travel can be viewed as elitist, given it is only available to a minority of students. Creating interactions across borders digitally by teaching a class with a team of teachers from two or more geographic locations can facilitate a more equitable exchange of different perspectives, with dialogue among participants creating global learning opportunities relevant to fulfilment of the SDGs. Digital arrangements are inexpensive, widely available, and can open minds even when airports are closed.

Before the pandemic, international partnerships typically entailed student and faculty exchanges and collaborative academic work including research, the latter supported by in-person workshops, symposia and conferences. However, COVID-19 showed us that many of the current in-person interactions could be done more easily and more efficiently via teleconferences (e.g. webinars or meetings with a large and diverse group of stakeholders from the private sector, citizens, mayors, etc.). Now, and going forward, as HE responds to the ongoing challenges of COVID-19, we will likely see a greater focus on shared learning by design across countries and national borders. Rather than months or even years to secure committee approvals, partnerships based on shared interests will support dialogue across cultures and borders in support of SDG 17. This may mean a very different kind of partnership arrangement, sharing information, research, credit, and even costs to better serve students, institutions and communities. When we are at a point where in-person convening is routine, adopting a mindset of reciprocity being locally rooted, but globally connected could be the model to play a critical role in the implementation of the SDGs. Institutions with overseas in-country delivery through a branch or satellite campus model or co-sharing with local providers may be at an advantage as we re-imagine internationalization.

### Teaching and learning

In a matter of weeks, US HE institutions largely transitioned to remote learning at a speed no one would have thought possible with all concerned doing their best and people tolerant of the trial and error nature of online encounters. For example, Tulane University had been talking about moving online for some 20-years and did so in a week, while Indiana University having worked for some 10-months on a plan to revise the homepage of their website delivered it in just two weeks. This reaction was not however a conscious shift to online learning and digital pedagogy, which is where the sector is now challenged to respond. While there is still a lot of uncertainty, it seems likely that even after the COVID-19 measures currently in place are lifted, the balance of face-to-face, blended and online learning at many universities and colleges will shift in the medium- if not long-term. Some educators feel more comfortable with traditional methods of delivery and do not see online delivery as a viable way of engaging students, a view held by many students and employers. Generational analysis conducted by Pearson in the U.K. (based on 2400 responses) indicated a growing preference for digital learning among Generation Z (those born between 1995 and 2015), and the increasing use of tools like YouTube [52]. However, the research also revealed Gen Z’s prefer learning paradigms that stress the social aspects of learning. The challenges to grow stakeholder confidence in online delivery and find learning models that advance equity for learners are significant as we re-imagine pedagogy for the 21st-century and the interdisciplinary approaches demanded by the SDGs.

As the pandemic revealed societal inequities, with mortality and morbidity levels correlated with the social determinants of health [53], so too opportunity gaps became more visible. Outwith issues relating to limited access to technology among lower income students, there is some evidence that the transition to online learning is not equitable in terms of learning opportunity. Faculty too need more support to make the shift to online delivery; simply transferring face-to-face delivery to online often results in a lower quality learning experience. The pedagogy required to deliver high-quality online courses are noticeably different to those needed to teach in a face-to-face setting, with additional training needed [54]. For example, creating and maintaining a strong presence online, educators have to overcompensate for the lack of physical proximity, ideally through regular and varied verbal communication and the use of non-verbal communication such as emoticons. Promoting reflection and dialogue through quality asynchronous discussion, faculty need to have the contributions from each learner monitored and facilitate discussions, demanding more support from teaching assistants. While there are clearly benefits to synchronous learning, this can put additional pressure on both students and educators in building inclusive online communities at course level to satisfy the need for the social aspects of learning, including peer learning [55], and using tools to support discussions among students such as breakout rooms or online exchanges with the professor. It has been shown that online learner motivation is related to the amount of interaction with their educators and the level of enthusiasm they convey, modelling a personalized presence and community culture that supports retention [42]. For those studying remotely, there will be new demands for pastoral and academic support, potentially in different time zones. Rather than simply trying to replicate an in-person experience, which we know can often be inadequate in advancing learning, digital pedagogy done well by skillful practitioners can present a cheaper more personalized learning experience that creates space for curiosity and global encounters [31].

The Unbundled University research project [56], funded by the U.K.’s Economic and Social Research Council and the National Research Foundation in South Africa, explored a range of issues in relation to the expansion of online education in universities in the two countries, including partnerships with private companies and the disaggregation of learning and teaching materials for delivery online (‘unbundling’). Highlighting the importance of having a clear online education strategy, with investment in faculty and staff skills, the project also noted the opportunity partnership with private companies and online platform providers can present to the creation of blended learning delivery. The pivot to online learning is here to stay. Pearson’s second Global Learner Survey [42] of over 7000 people from seven countries conducted in June 2020 in the midst of the COVID-19 pandemic revealed more than three-quarters of respondents considered online learning will be part of the HE experience going forward, with concerns about affordability and safety driving student choices for Fall 2020 [31, 41].

Even before COVID-19, decision makers in HE were grappling with complex discussions in relation to campus-based and online education, and experimenting with growing their online education portfolio through Massive Open Online Courses (MOOCs) etc. Few however had turned this into a revenue generating activity, and fewer still have experience of delivering successful large-scale fully online degrees, for example the Harvard School of Continuing Education (https://www.extension.harvard.edu) offers undergraduate, graduate, and pre-medical studies along with programs for professional development and learning in retirement. In this case, the approach allowed Harvard to offer a more flexible education and adapt the courses to societal needs, including SDG-relevant topics as demonstrated in the Sustainability Degree Program (https://www.extension.harvard.edu/academics/graduate-degrees/sustainability-degree). Leaving the transition to busy faculty is not enough, and HE needs to invest in scaling up online education, bringing in instructional designers, video producers and content writers, all without eroding academic autonomy, control over teaching materials and pedagogical approaches and cementing the crucial role of academic teachers as navigators of learning journeys. The COVID-19 crisis has fueled a sector-wide pivot to online learning that should not be seen as a makeshift emergency reaction, rather it can part of a conscious response to advancing sophisticated high-touch digital experiences tailored to advance personalized learning and close the opportunity gap helping foster the implementation of the SDGs.

### Employability

COVID-19 is already impacting on graduate recruitment into the workforce, reducing the number of graduates being hired this year by large firms and small- and medium-sized enterprises (SMEs) [57] producing some forbidding scenarios for post-crisis levels of global unemployment. McKinsey recently called COVID-19 the “biggest destroyer of jobs, ever” [58] noting 195 million jobs were lost in Q2 of 2020 alone, with two service industries (accommodation/food; wholesale/retail) accounting for 40% of at risk jobs with 80% of customer service/sales jobs at risk. This has had a disproportionate impact on minorities and marginalized communities, increasing existing social cleavages. In the short-term, employers will delay or cancel normal graduate recruitment; in the medium-term, graduates will operate in a more competitive labor market; and in the long-term, this year’s students will be graduating at the start of a recession that could last for some time. While some in HE disparage ‘the employability agenda’, the sector has largely embraced it, with many competing to pronounce themselves as the most likely to get you a graduate job. The employment imperative has increased among degree seekers from around one in two to over 90% in recent years, with COVID-19 likely to super-charge this trend [42]. Much of the HE sector’s growth has been bound up with human capital theory and the promise that more education means more skills, which equates to a better job with more money; the pandemic is causing us to revisit this model as well as inviting us to revisit the types of skills needed.

When faced with an economic crisis, historically people have turned to education as a way to better prepare for the future. With COVID-19, it is yet unclear whether this will be the prevailing trend. The Strada Public Viewpoint Survey on COVID-19 and its impact on work and education [41], with responses from over 5000 adults, reported that the number of people intending to pursue education or training has not increased. Those that are considering a course in the next five years are looking at different providers than before the pandemic, such as trade schools, online-only programs and community colleges—preferring non-degree programs and courses that would help them reskill or upskill in line with the new economy and new jobs that will be created. Among the findings, the survey reports that some 28 million US adults (11% of Americans) have cancelled their education plans because of COVID-19 and more report disruption to their plans. Universities and colleges need to ensure the Class of 2020 do not become a lost generation [59, 60], and mobilize support for employability through their career services, as well as extending access to mental health support and professional education. The ‘crisis cohort’ who graduated in 2008 continue to be at a disadvantage up to a decade later [60], and there is little reason to suggest the current crisis will be any different. Employability provision has adopted several, often problematic, assumptions that have privileged individualism, competition and global mobility. In the world after this pandemic, employability may need to emphasize social justice, community and relationship building to foster collaboration and the contribution that people can make to their locality and to solve wicked problems rather than stressing high salary and high status roles.

### Sustainability

Reacting to the pandemic, HE institutions were largely in panic mode with little attention paid to their wider role as critical engines of change, helping people, planet and prosperity advance sustainable development. As the immediacy subsided, many universities and colleges responded by leaning into the crisis to apply their knowledge firepower as trusted sources of authority to help. From testing kits at the University of East Anglia [23], to the University of South Florida 3D-printing personal protective equipment [61] and vaccine development at the University of Cambridge [22], HE institutions continue to be at the forefront of the fight against the virus. It was inspiring to see the speed, scale and flexibility with which HE harnessed science and research, mobilized medical and health students and supported their communities locally and globally. While it is too early to assess the full impact of the pandemic on public health, the economy and society, the HE sector is a critical contributor to finding solutions and needs to act more boldly and collaboratively to demonstrate the value it delivers.

Drawing on the 17 SDGs, the Times Higher Education (THE) Global Impact Rankings [62] offers a proxy measure of the extent to which HE has positive social and economic impacts on the planet; the THE ranking is not however without criticism in terms of the indicators used and methodology adopted [63, 64]. It is the case however that the THE ranking demonstrates impact ranging from action on climate change to gender equality, good health and wellbeing and is the first such model to use such criteria, ranking HE institutions from the developed world to the lowest-income countries and regions. From 89 countries and regions across six continents, 859 universities were ranked for at least one SDG, and 766 included in the overall ranking; many of the top 100 places are held by universities from countries and regions that have not appeared previously in the upper quartile of the THE world rankings—for instance Iran, Indonesia, Malaysia and Mexico [62].

A fundamental challenge to the sustainability of HE across so many levels, from the business model through to its social contract, COVID-19 represents a ‘teachable moment’ for the sector to accelerate sustainability, paying greater attention to the trade-offs and dilemmas presented by its activities and its contribution to the fulfilment of the SDGs. HE can use its research to inform and influence partners in their communities and wider networks, closing knowledge gaps as well as skills gaps, being in an exceptional position not only to create knowledge but also to inspire action. This requires will and wherewithal, but COVID-19 has shown that HE institutions have the ability, power and agility to do that. The sector can build on existing initiatives, such as the Sustainable Development Solutions Network (SDSN, https://www.unsdsn.org), which is promoting integrated approaches to implementing the SDGs and the Paris Agreement on Climate Change through education, research, policy analysis, and global cooperation. The SDSN published a tool to get help HE engage with the SDGs [65] that is now being complemented with a specific guide on Teaching and Learning [66]. In the U.K., the EAUC ‘The Alliance for Sustainability Leadership in Education’ (https://www.eauc.org.uk/) is working with GuildHE (https://guildhe.ac.uk/), the Association of Colleges (https://www.aoc.co.uk/) and Universities U.K. (https://www.universitiesuk.ac.uk/) to galvanize sector-wide leadership around climate change and sustainability via the Climate Commission [66] that was set up to provide direction, leadership and consensus on confronting climate change.

What should focus attention and mobilize HE leaders once the COVID-19 situation is managed are the wider gamut of systemic risks to our populations and planet. Whatever the cause of the current pandemic, living within our means and in balance with the natural world will be reinforced by global, national and local recovery priorities. These need to be reflected within HE programs and research. Because of COVID-19, the concept of a systemic existential threat is front of mind for all people, with the pandemic threatening lives now and shaping a next normal. The collective global urgency harnessed to tackle COVID-19 might be contrasted unfavorably with that needed to address climate change, but the pandemic shows us that we have an inherent leadership and change capacity once we gather humanity around the problem and work collectively with a global mindset.

### Multi-stakeholder partnerships

The response to a crisis as complex as COVID-19 requires a multisectoral and multidimensional approach, covering areas such as health, mobility, logistics, protection, and developing innovations in technology, business models, regulation, or policymaking [67]. Institutions of HE have a range of knowledge assets that may be oriented to tackle the pandemic, bringing together a wide range of disciplines and research facilities including innovation hubs. They also bring other valuable but intangible assets such as neutrality, legitimacy, convening authority and connection with the youth. While HE has enjoyed close connections with other societal stakeholders (public sector, companies, and civil society), typically these have been somewhat atomized and bi-lateral [68]. The shared purpose represented by the COVID-19 crisis is an opportunity to develop deep and trustful multi-stakeholder collaborations [69], that can help address the societal challenges and contribute to the implementation of the SDGs in the wake of the pandemic. Interacting in diverse, flexible and reciprocal collaborative arrangements enables HE to work at scale to advance knowledge sharing and create impact [70].

By adopting a new collaborative mindset, the HE sector can be viewed as an important societal architect, bringing its unique assets into partnership with aligned multi-stakeholders. For example, innovative training programs devoted to connecting traditional knowledge with community-based needs, fostering students’ interactions with other agents to prepare them for a different employability future. Private companies, public sector bodies and civil society are called upon to play an active and interconnected role in furthering relationships with the HE sector. Establishing radical partnerships is a strategic imperative for sustaining HE institutions and pursuing the SDGs. Classical structures and ways of working however will not be able to fulfill the function of creating intermediary spaces to collaborate [29]. There is a need for structural innovations that may create a collaborative context, inter-disciplinary roadmap design and intra and inter organizational connections [71].

## Conclusions

The health, social and economic impact of the pandemic represent disruptive forces that are transforming the HE sector at pace and scale. While the majority of US HE institutions were exposed to the shocks caused by, or accelerated by the COVID-19 crisis, each was variously impacted dependent on their unique assets, positioning and risk appetite. Some are weathering the storm, others may go to the wall and some will emerge stronger, leaner and more agile. With a greater awareness of their strengths and weaknesses, we foresee a more collaborative mindset and a renewed focus on equity and a deeper appreciation of the centrality of HE to the knowledge economy. The pandemic can be an important moment for HE to demonstrate that what it does matters fundamentally to a civil society. It is here that the SDGs can influence profoundly the ‘next normal’ for the HE sector with a focus on fulfilment of Agenda 2030 [16].

Here, we considered how the sector *reacted* to the pandemic crisis and then went on to *respond* more deliberatively and consciously. The sector is now moving forward to *reimagine* and *renew* itself, with the SDGs representing a shared purpose for the sector at large and the stakeholder community represented by an individual institution. In this way, we anticipate HE will play a fuller role in addressing the societal challenges of our day and support resilience of the community it serves. Rather than fighting for a sense of normalcy, we urge the sector to accept that HE is forever changed and embrace this moment of punctuated equilibrium to advance HE’s profound transformational impact on people, prosperity and planet, and its material contribution to fulfilment of the SDGs.

Fundamentally, while all universities and colleges are unique given their history, mission and activities of faculty, staff and students, they also share a focus on enabling learning and supporting scholarship [72]. Here, while our convenience sample was from one US state and captured a range of different institutional archetypes, it did not include major public or private research universities. Given that, the insights offered by the seven university/college presidents did serve to reflect a diversity of mission, history, academic specialisms and student profiles [73]. The external pressures to change brought by the COVID-19 pandemic exert a pressure on an organization to adjust. In this case, HE with each institution differentially affected based on their resilience across a range of fronts from leadership through to partnership as discussed. In addition, wider societal demands and expectations will influence what they prioritize and the innovation they choose to pursue. Our early view is that differentiation will continue, with a more heterogenous global sector emerging – albeit with a more widespread sector engagement around the SDGs given the ‘humanness’ of the pandemic as a disruptive force.

Higher education is an enduring social good, transforming lives and an essential driver of equity and economic prosperity. While the pandemic served to both accelerate underlying global trends and reveal social inequities in the sector, it also served to advance the importance of a knowledge-led society and the value of experts offering us an opportunity to reclaim HE’s moral purpose. As those in HE reacted to the crisis, some valuable innovations will fuel its response over the longer-term and contribute to implementing the SDGs, as summarized in Table 1. Recognizing that we will be living *with* COVID-19 once the pandemic subsides, HE will need to prioritize the health and well-being of in-person instruction and campus life alongside investments in sustaining online learning and re-creating a dispersed scholarly community. We propose that convening students, staff and faculty around a shared purpose as represented by the SDGs can serve to both navigate the disruption and secure the long-term health of the HE sector.

COVID-19 is a moment of crisis for HE, re-shaping the sector in real-time and placing unprecedented demands on all stakeholders. Accelerating pre-COVID trends in the sector, in particular a focus on sustainability and equity, the pandemic represents an opportunity to make HE better. The sector went into the crisis with a range of pressing strategic and macro-economic challenges, accompanied by growing public unease about affordability and impact. Seeking to navigate these volatile, uncertain, complex and ambiguous times—so-called VUCA conditions—sustainability can be positioned as a strategic response to the pandemic and a major driver of HE’s future, helping the sector reimagine itself in line with the SDGs. HE is not a monolithic sector, nor are the institutions involved uniformly positioned to thrive. However, sustainable HE demands a new social contract is forged with society, with sustainability a driver of radical transformation. By acting to protect HE over the long-term, the sector can re-imagine itself and sustain shared value, making its fullest contribution to a world where *no*-*one will be left behind* [74].

## Data Availability

Not applicable.

## Footnotes

1 College and University Presidents interviewed by Wendy Purcell were: Helen Drinan, Simmons University; Paula Johnson, Wellesley College; Toni Hays, Regis College; Deborah Jackson, Cambridge College; Yves Solomon-Fernandez, Greenfield Community College; Valerie Roberson, Roxbury Community College; Alison Davis-Blake, Bentley University.
